# Complementary primary mental health programs for young people in Australia: Access to Allied Psychological Services (ATAPS) and *headspace*

**DOI:** 10.1186/s13033-017-0125-7

**Published:** 2017-02-10

**Authors:** Bridget Bassilios, Nicolas Telford, Debra Rickwood, Matthew J. Spittal, Jane Pirkis

**Affiliations:** 1 Centre for Mental Health, Melbourne School of Population and Global Health, The University of Melbourne, Melbourne, VIC 3010 Australia; 2 School of Public Health, The University of Queensland, Herston, QLD 4006 Australia; 3 headspace National Youth Mental Health Foundation, Melbourne, VIC Australia; 4 Faculty of Health, The University of Canberra, Canberra, ACT Australia

**Keywords:** Young people, Youth, Adolescents, Mental health services, Primary health care, Mental health policy

## Abstract

**Objective:**

Access to Allied Psychological Services (ATAPS) was introduced in 2001 by the Australian Government to provide evidence-based psychological interventions for people with high prevalence disorders. *headspace*, Australia’s National Youth Mental Health Foundation, was established in 2006 to promote and facilitate improvements in the mental health, social wellbeing and economic participation of young people aged 12–25 years. Both programs provided free or low cost psychological services. This paper aims to describe the uptake of psychological services by people aged 12–25 years via ATAPS and *headspace*, the characteristics of these clients, the types of services received and preliminary client outcomes achieved.

**Methods:**

Data from 1 July 2009 to 30 June 2012 were sourced from the respective national web-based minimum datasets used for routine data collection in ATAPS and *headspace*.

**Results:**

In total, 20,156 and 17,337 young people accessed two or more psychological services via ATAPS and *headspace*, respectively, in the 3-year analysis period. There were notable differences between the clients of, and the services delivered by, the programs. ATAPS clients were less likely to be male (31 vs 39%) and to reside in major cities (51 vs 62%) than *headspace* clients; ATAPS clients were also older (18–21 vs 15–17 years modal age group). There was some variation in the number and types of psychological sessions that young people received via the programs but the majority received at least one session of cognitive behavioural therapy. Based on limited available outcome data, both programs appear to have produced improvements in clients’ mental health; specifically, psychological distress as assessed by the Kessler-10 (K-10) was reduced.

**Conclusions:**

ATAPS and *headspace* have delivered free or low-cost psychological services to 12–25 year olds with somewhat different characteristics. Both programs have had promising effects on mental health. ATAPS and *headspace* have operated in a complementary fashion to fill a service gap for young people.

## Background

Youth mental illness is an important public health problem in Australia and internationally. Early identification of children and young people at risk of mental illness creates opportunities for intervention that can help to avert or ameliorate problems in later life. Primary mental health care services have an important role to play in recognising vulnerable individuals and offering appropriate care and support.

### Youth mental health

A series of surveys conducted in Australia, which have considered high prevalence disorders across the age spectrum, can shed light on the extent of mental health problems in young people. The most recent National Surveys of Mental Health and Wellbeing (NSMHWB), conducted in 2007, recruited 8841 individuals aged 16–85 years [1]. Findings indicated that among those aged 16–24 years, 23% males and 30% females (26% overall) had experienced anxiety, affective or substance use disorders in the previous 12 months [2], compared to 20% of the overall sample surveyed [1]. The most recent survey of child and adolescent mental health, conducted in 2013–14, recruited 6310 parents and carers of children aged 4–17 years and 2967 children aged 11–17 years and found that 14% of children and adolescents had a mental disorder (most commonly attention-deficit/hyperactivity disorder followed by anxiety disorders) in the previous year, which was associated with a substantial number of days absent from school [3].

These surveys also show that young people with mental health problems tend not to access services. The adult survey reported that less than 25% of people aged 16–24 years with mental disorders accessed services in the previous 12 months [2]. The 2013–14 child and adolescent survey reported 56% of 4–17 year olds with mental disorders had accessed mental health services (e.g., family doctors, psychologists, paediatricians and counsellors/family therapists) in the year preceding the survey [4], which was an improvement in the rate of service use reported in 1997; 25% in the preceding 6 months [5]. Furthermore, the adult survey found gender differences in service use among those aged 16–24 years, with only 13% of males and 31% of females with mental disorders accessing any mental health service [6]. Even when young people do seek help, there is often a significant delay between onset of symptoms and accessing services, which varies according to type of disorder, gender, population group and geographic location [7]. Personal factors (e.g., stigma and negative attitudes to, and experiences of, treatment) and structural barriers (e.g., location, cost and availability of services) contribute to treatment delays [8].

The World Mental Health (WMH) Survey Initiative, which encompasses the Australian NSWMHB, is a project of the World Health Organization (WHO) which aims to obtain epidemiological data on mental, substance and behavioural disorders in all WHO regions [9]. The WMH Survey Initiative found that in 17 countries in Africa, Asia, the Americas, Europe and the Middle East, the median and inter-quartile (IQR) age of onset is very early for some anxiety disorders (7–14, IQR 8–11) and impulse control disorders (7–15, IQR 11–12) [9]. Given that many mental disorders begin in childhood or adolescence, access to services that focus on early identification and treatment may mitigate the persistence or severity of primary disorders and prevent comorbid disorders [9].

### Primary mental health care in Australia

In response to the public health problem of youth mental ill-health, the Australian Government introduced a suite of reforms to improve access to mental health care for young people, either by targeting them directly or by offering care across the age range.

The Access to Allied Psychological Services (ATAPS) program, funded by the Australian Government from July 2001 to June 2016, provided primary care for common mental disorders across the lifespan. ATAPS enabled general practitioners (GPs) to refer patients with high prevalence disorders (e.g., depression and anxiety) to mental health professionals (predominantly psychologists) for free or low-cost, evidence-based mental health care (most commonly cognitive behavioral therapy, or CBT). This care was typically delivered in up to 12 (or 18 in exceptional circumstances) individual and up to 12 group sessions per calendar year. Review by the referring GP was essential after each block of six sessions and/or the final session [10]. Over time ATAPS evolved to offer more flexible services to particular at-risk and/or hard to reach populations [11]. Nationwide, ATAPS has been delivered through regionally-based primary health care organisations: specifically, these have included 31 Primary Health Networks since July 2015, previously 61 Medicare Locals (July 2011–June 2015) and originally over 100 Divisions of General Practice (July 2001–June 2010).

ATAPS has been independently evaluated since its introduction, with findings indicating high program uptake by close to 280,000 clients [12] in both urban and rural areas [13, 14] and positive outcomes for clients [15] and providers [16]. The introduction of the continuing Better Access to Psychiatrists, Psychologists and GPs (Better Access) initiative in 2006 influenced the nature and direction of ATAPS. Better access facilitates similar access to primary mental health care via fee-for-service rebates under Medicare, Australia’s publicly funded universal health care system, operated by the government authority Medicare Australia [17]; however, unlike ATAPS, its funding is uncapped. Consequently, following the introduction of Better Access, ATAPS refined its focus to offer more flexible services to particular at-risk populations (e.g., people at risk of suicide, people affected by extreme climatic events, children with mental disorders) that were not available via either the original form of ATAPS, which operated simultaneously, or via Better Access.

*headspace* National Youth Mental Health Foundation Ltd (*headspace*), another Australian Government initiative, introduced in 2006, specifically targeted young people with mental health issues [18]. *headspace* aimed to reorient the service system to create highly accessible, youth-friendly, integrated service hubs and networks that provide free or low-cost evidence-based interventions and support to young people aged 12–25 years [18]. Each local *headspace* centre was directed by a lead agency on behalf of a local partnership of organisations responsible for providing more integrated and coordinated responses for young people across primary care, mental health, alcohol and other drugs, and social, educational and vocational issues. The key aim was to improve mental health outcomes for young people through greater access and engagement, earlier intervention, more holistic care, and better service integration [19]. Between 2006 and July 2014, 67 *headspace* centres were opened and services were provided to almost 125,000 young people in metropolitan, regional and rural/remote areas across Australia. Bipartisan government support meant that the number of *headspac*e centres steadily increased, with around 85 centres established by early 2015, and plans to increase to up to 110 centres across Australia in 2016/17 [20]. The psychological services delivered by *headspace* were often funded via the Better Access program (57.4%) and infrequently via ATAPS (7.8%) [21].

As a result of a major review of all Australian mental health services completed in 2014 [22], the mental health system is undergoing major reform. The key finding of this review was that Australia’s mental health system is poorly planned and integrated resulting in less than optimal wellbeing and participation, therefore hindering productivity and economic growth. Consequently, recommendations emerged to improve mental health system sustainability based on three key principles: person-centred design in which services are organised around the needs of people, a new system architecture based on a stepped care framework that provides services of varying intensity to match people’s level of need, and shifting funding to more efficient and effective ‘upstream’ services and supports (i.e. population health, prevention, early intervention, recovery and participation) [22]. To this end, from July 2016, Primary Health Networks became the commissioners of primary care psychological treatment (including both ATAPS and *headspace* amongst other programs) within a stepped care approach according to local population mental health needs [23].

ATAPS and *headspace* have differed not only in terms of who they targeted and how their services were delivered, but also in terms of what they offered. Both ATAPS and *headspace* provided psychological services and had an overlapping client group but *headspace* provided additional health and biopsychosocial services (including physical and sexual health, alcohol or other drug, and vocational services) as well as community awareness and youth engagement activities. Although both programs have separately examined service delivery for young people across different time periods [19, 24], to date a comparison between the programs and the young people to whom services have been delivered has not been undertaken. Therefore, we examined the uptake of both programs by clients aged 12–25 years, the characteristics of these clients, the types of services delivered and the mental health outcomes produced.

## Methods

### Data sources

We used ATAPS data from a web-based dataset, which forms a national minimum dataset used for research and evaluation. Data were routinely collected via project officers or providers who entered de-identified client data that they tracked using unique identifier codes into the web-based interface. We extracted from the minimum dataset data on the number of clients and their socio-demographic (e.g., age, gender, level of income) and clinical (e.g., diagnosis, previous psychiatric service use, clinical outcome) characteristics; and the number, type and duration of sessions, and the nature of the interventions provided. ATAPS data were extracted from the minimum dataset on 29 October 2012.

Similarly, we used *headspace* administrative data that were routinely collected and entered by providers into the *headspace* minimum dataset, which captured data on client characteristics, occasions of service (‘sessions’) and client outcomes through an electronic record. Data items included in the analysis period were routinely collected for each client at assessment, throughout the duration of care and at closure. Each week, data were automatically stripped of identifying client and clinician information, extracted to the *headspace* national office data warehouse and used for the purpose of monitoring and evaluation. A review in 2012 resulted in a major shift in how data were collected across the centres, and a new system was implemented in 2013 to improve compliance and the ability of the service to measure and assess service outcomes [20]. *headspace* data were extracted from the minimum dataset on 21 November 2013.

The variables captured in the ATAPS and *headspace* minimum datasets that were comparable are shown in Table 1. Both ATAPS and *headspace* datasets captured client postcodes at the time of referral, which enabled us to classify region of residence according to the Australian Standard Geographical Classification (ASGC) Remoteness Structure. The ASGC classifies geographical areas into six categories (the sixth of which was not relevant for our purposes): (1) Major cities; (2) Inner regional; (3) Outer regional; (4) Remote; (5) Very remote; and (6) Migratory [25]. Australian Bureau of Statistics mapping files which provide the proportion of the Australian population within a given postcode allocated to a particular remoteness area were used to calculate the proportion of clients to be assigned to the five remoteness areas. ASGC remoteness classifications were produced using 2011 mapping [25].

**Table 1:** Variables captured in the ATAPS and *headspace* minimum datasets

Variable	ATAPS	*headspace*
Sociodemographic characteristics		
Gender	√	√
Age	√	√
Indigeneity	√	√
Postcode	√	√
Low income^a^	√	√
Clinical characteristics		
Previous history of mental health care^b^	√	√
Diagnosis	√	
Stage of illness^c^		√
Treatment received		
Number of sessions^d^	√	√
Treatment received^e^	√	√
Client copayment	√	
Client outcomes		
K-10	√	√
Other measures	√	

*ATAPS* Access to Allied Psychological Services

^a^Low income was determined according to the GP’s judgement in ATAPS and by receiving a government benefit via *headspace*

^b^There was relatively poor compliance with the completion of ‘previous history of mental health care’ in *headspace*

^c^Stage 1: mild or increased risk; Stage 2: moderate or sub-threshold; Stage 3: severe or full threshold

^d^ *headspac*e delivers both psychological and non-psychological services. Only sessions in which psychological services were delivered were analysed and may be somewhat over-represented as multiple psychological services delivered in a single session are recorded as multiple services/sessions

^e^Treatments explicitly involving behavioural interventions and/or cognitive interventions were classified as CBT; assessment, psycho-education, relaxation strategies, skills training and/or interpersonal therapy were classified as non-CBT

### Inclusion criteria

ATAPS and *headspace* clients were included in the analyses if they received two or more sessions of psychological care between 1 July 2009 and 30 June 2012. Psychological care is defined as evidence-based talking therapy delivered by mental health professionals such as psychologists, social workers, mental health nurses, occupational therapists and Aboriginal and Torres Strait Islander health workers. This timeframe was selected as it provided the longest duration of comparable and recent data for the two programs in that it pre-dated the transition of data to Medicare Locals for ATAPS (which occurred from October 2012 to March 2013) and the change in the *headspace* data capture system (in 2013). Noting that *headspace* services were not limited to psychological care, only *headspace* sessions in which psychological services were delivered were analysed. This excluded sessions that mainly comprised assessment and engagement, and situational, alcohol and other drug, vocational and psychosocial issues. Two or more sessions of psychological care were deemed necessary in order to enable analysis of standardised outcome scores; that is, a single session providing a single outcome score would not provide information on change in symptoms.

ATAPS clients were included if they were aged between 12 and 25 years at the time of referral; all *headspace* clients were in this age bracket. Only *headspace* clients from the 30 fully operational centres during the data analysis period were included; a further 10 centres opened during this period but were not operational for the entire analysis period [26]. Given the 3-year duration of the analysis period, a minority of clients across the programs (4% for ATAPS and unavailable for *headspace*) received more than one episode of care, meaning they were treated either for the same presenting issue on multiple occasions, each with distinct start and end dates, or for more than one presenting issue. Treatment and outcome data were analysed for each client’s first episode of care during the analysis period.

Outcome data were not available for the full sample from ATAPS or *headspace*. The sub-samples included in the analysis of outcomes were those for whom pre- and post-treatment Kessler-10 (K-10) [27] outcome data were available on the basis that this was the most commonly used measure by the treating practitioner in ATAPS and the only comparable measure available during this period in the *headspace* data. Furthermore, *headspace* K-10 time one data were collected at intake and time two data were derived from either a review or discharge session; that is, during the course of, or at the completion of treatment. The K-10 is a 10-item self-report measure of non-specific psychological distress in the previous 30 days, with sound psychometric properties [27]. A sample item is ‘During the last 30 days, about how often did you feel hopeless?’ Each item is rated on a five-point scale ranging from 1 (none of the time) to 5 (all of the time) with total scores from 10 to 15 indicating low, 16–21 moderate, 22–29 high and 30–50 very high distress. Clients who did not have a ‘matched pair’ of pre- and post-treatment scores, or who were assessed using other outcomes measures, were excluded.

### Statistical analyses

We calculated the proportion of ATAPS and *headspace* clients who had used the respective services by client (gender, age, Indigeneity, remoteness area, income, previous history of mental health care) and session (number of sessions, type of intervention) characteristics. Two-sample tests of proportions were used to determine if the difference in proportions between these programs was significantly different from zero (differences greater than zero signify that the proportions differ between the two programs after accounting for sampling variation).

We also examined K-10 outcome data for each program. Specifically, we examined the mean difference in the pre- and post-treatment K-10 scores and analysed the proportion of clients with low, moderate, high and very high psychological distress at pre- and post-treatment for each program.

Stata 13.1 was used to conduct the two-sample tests of proportions and all other analyses were conducted using SPSS v.21.

## Results

### Uptake of ATAPS and *headspace*

Between 1 July 2009 and 30 June 2012, a total of 20,156 ATAPS and 17,337 *headspace* clients aged 12–25 years met the criteria for inclusion in the analyses. ATAPS clients were offered 110,251 sessions, 9.6% of which were unattended. *headspace* clients received 117,423 psychological ‘services’. The number of *headspace* services is not directly comparable with the number of ATAPS sessions because multiple interventions delivered within a single *headspace* session could be counted as more than one ‘service’ and non-attendance data were unavailable for *headspace*.

### Characteristics of all clients in receipt of sessions, and their treatment profile

Table 2 shows the socio-demographic, clinical and treatment characteristics of all young people, and those with available outcome data, who received services via ATAPS and *headspace*, displaying the proportions, differences between proportions and associated confidence intervals. Overall, the majority of clients were female for both programs, however, a greater proportion of males accessed *headspace* than ATAPS (39 vs 31%, p < 0.0001). Young people accessing ATAPS were more likely to be older (18–21 vs 15–17 years modal age group) and to be on a low income (67 vs 23%, p < 0.0001) than those accessing *headspace*. Similar proportions of ATAPS and *headspace* clients were Indigenous (5%). ATAPS clients were less likely to reside in major cities (51 vs 62%, p < 0.0001) and slightly more likely to reside in outer regional/remote/very remote locations (17 vs 14%, p < 0.0001) than *headspace* clients. The majority of ATAPS clients had not previously received psychiatric care (44%). While data on previous receipt of psychiatric care were not available for the majority of *headspace* clients (82%), for those with data, just over half had not previously received psychiatric care (53%). Although it appears that proportionally more *headspace* than ATAPS clients were represented across the baseline K-10 severity categories, it should be noted that this information was not available for 70% of ATAPS (many of whom may have been assessed using measures other than the K-10) and 53% of *headspace* clients, respectively.

**Table 2:** Socio-demographic, clinical and treatment profiles of all ATAPS and *headspace* clients, and those with pre- and post-treatment outcomes, July 2009 to June 2012

	All ATAPS (N = 20,156)		ATAPS with pre- and post treatment outcomes (N = 1303)		All *headspace* (N = 17,337)		*headspace* with pre- and post treatment outcomes (N = 1326)		Difference between all ATAPS and all *headspace*	
									Percent difference (95% CI)	p value
	n	%	n	%	n	%	n	%		
Socio-demographic characteristics										
Gender										
Male	6250	31.0	363	27.9	6741	38.9	530	40.0	−7.9 (−6.9 to −8.9)	<0.0001
Female	13,906	69.0	940	72.1	10,596	61.1	796	60.0	7.9 (6.9 to 8.9)	<0.0001
Age (years)										
12–14	2801	13.9	149	11.4	3356	19.4	295	22.2	−5.5 (−4.7 to −6.3)	<0.0001
15–17	4657	23.1	319	24.5	6406	36.9	509	38.4	−13.8 (−12.9 to −14.7)	<0.0001
18–21	6584	32.7	422	32.4	5034	29.0	347	26.2	3.7 (2.8 to 4.6)	<0.0001
22–25	6114	30.3	413	31.7	2541	14.7	175	13.2	15.6 (14.8 to 16.4)	<0.0001
Indigeneity										
Indigenous	941	4.7	41	3.1	885	5.1	49	3.7	−0.04 (−0.004 to −0.08)	0.0732
Not indigenous	15,842	78.6	1135	87.1	14,213	82.0	1158	87.3	−3.4 (−2.6 to −4.2)	<0.0001
Unknown	3373	16.7	127	9.7	2239	12.9	119	9.0	3.8 (3.1 to 4.5)	<0.0001
Remoteness area										
Major city	10,190	50.6	795	61.0	10,683	61.6	831	62.7	−11.0 (−10.0 to −12.0)	<0.0001
Inner regional	5265	26.1	371	28.5	4075	23.5	385	29.0	2.6 (1.7 to 3.5)	<0.0001
Outer regional/remote/very remote	3391	16.8	99	7.6	2506	14.5	108	8.1	2.3 (1.6 to 3.0)	<0.0001
Unknown	1260	6.3	38	2.9	73	0.4	2	.2	5.9 (5.5 to 6.2)	<0.0001
Income^a^										
Low income	13,435	66.7	908	69.7	3936	22.7	291	21.9	44.0 (43.1 to 45.0)	<0.0001
Not low income	2514	12.5	203	15.6	8667	50.0	721	54.4	−37.5 (−36.6 to −38.4)	<0.0001
Unknown	4207	20.9	192	14.7	3111	17.9	265	20.0	3.0 (2.2 to 3.8)	<0.0001
Clinical characteristics										
Previous history of mental health care										
No previous history of mental health care	8822	43.8	589	45.2	1689	9.7	244	18.4	34.1 (33.3 to 35.0)	<0.0001
Previous history of mental health care	6833	33.9	506	38.8	1481	8.5	202	15.2	25.4 (24.6 to 26.2)	<0.0001
Unknown	4501	22.3	208	16.0	14167	81.7	880	66.4	−59.4 (−58.6 to −60.4)	<0.0001
Pre-treatment K-10										
Low (10–15)	248	1.2	62	4.8	733	4.2	90	6.8	−3.0 (−2.7 to −3.3)	<0.0001
Moderate (16–21)	590	2.9	119	9.1	1279	7.4	187	14.1	−4.5 (−4.0 to −5.0)	<0.0001
High (22–29)	1790	8.9	411	31.5	2365	13.6	411	31.0	−4.7 (−4.1 to −5.3)	<0.0001
Very high (30–50)	3497	17.3	711	54.6	3858	22.3	638	48.1	−5.0 (−4.2 to −5.8)	<0.0001
Unknown	14,031	69.6	–	–	9102	52.5	–	–	17.1 (16.1 to 18.1)	<0.0001
Treatment profile										
Number of psychological sessions										
2–3	6370	31.6	136	10.4	6584	38.0	258	19.5	−6.4 (−5.4 to −7.4)	<0.0001
4–5	4713	23.4	202	15.5	3669	21.2	244	18.4	2.2 (1.4 to 3.0)	<0.0001
6	4455	22.1	461	35.4	1313	7.6	150	11.3	14.5 (13.8 to 15.2)	<0.0001
7–12	3876	19.2	414	31.8	3660	21.1	418	31.5	−1.9 (−1.1 to −2.7)	<0.0001
13–18	742	3.7	90	6.9	2111	12.2	256	19.3	−8.5 (−7.9 to −9.0)	<0.0001
Treatment received										
Received CBT in at least one session^b^	13525	67.1	981	75.3	10,110	58.3	1045	78.8	8.8 (7.8 to 9.7)	<0.0001

*ATAPS* Access to Allied Psychological Services, *CBT* cognitive behavioural therapy

^a^Low income is determined according to GP judgement in ATAPS and by being in receipt of a government benefit in *headspace*

^b^Includes behavioural interventions and/or cognitive interventions, with or without diagnostic assessment, psycho-education, relaxation strategies, skills training and/or interpersonal therapy

The majority of ATAPS clients had a diagnosis of depression (30%), depression with co-morbid anxiety (26%) and anxiety (18%); these diagnoses occurred in isolation or comorbidly with alcohol and drug use disorders, psychotic disorders and/or unexplained somatic disorders. Around 54% of *headspace* clients had stage of illness recorded; of these 51% were classed as mild or increased risk (Stage 1), 32% as moderate or sub-threshold (Stage 2) and 17% as severe or full threshold (Stage 3).

Despite differences in the recording of uptake of sessions between the programs, ATAPS clients were more likely to receive six sessions (22 vs 8%, p < 0.0001) and *headspace* clients were more likely to receive 2–3 and 13–18 sessions (38 vs 32%, p < 0.0001 and 12 vs 4%, p < 0.0001, respectively). *headspace* clients were less likely to receive strictly defined CBT in at least one session (58 vs 67%; p < 0.0001). However, findings in relation to number of sessions and type of treatment need to be interpreted in the context that within *headspace* clinicians were required to select the type of psychological service provided from a list of 30 possible mental health interventions, multiple interventions could be delivered and counted within a single service, and clients did not just receive mental health care but receipt of mental health care was a condition for inclusion in the study and only sessions devoted to mental health care were analysed. Although both programs offered a free or low-cost service, related data were recorded in the ATAPS, but not the *headspace*, minimum dataset. Only 11% of ATAPS clients paid a small fee in at least one session.

### Characteristics of clients with outcome data, and their treatment profile

Of all the ATAPS clients aged 12–25, around 6.5% (n = 1303) were included in the analyses of K-10 outcome data. Similarly, 7.6% (n = 1326) *headspace* clients were included in the analyses of K-10 data on the basis of having at least one follow-up K-10 score available at review or discharge.

As shown in Table 2, in the main, the profiles of ATAPS clients with outcome data were similar to the overall ATAPS sample included in the analyses. The exceptions were that those with outcome data were: somewhat less likely to be Indigenous (3 vs 5%) and reside in outer regional, remote or very remote locations (8 vs 17%); and somewhat more likely to be diagnosed with comorbid depression and anxiety (32 vs 26%), have received six or more sessions (74 vs 45%), have received CBT (75 vs 67%) and to have paid a copayment (19 vs 11%). Similarly, in the main, the profiles of *headspace* clients with outcome data were similar to the overall *headspace* sample included in the analyses with the exception that those with outcome data were: somewhat less likely to reside in outer regional/remote/very remote locations (8 vs 14%); and somewhat more likely to reside in inner regional locations (29 vs 23%), not be in receipt of low incomes (or be from low income families; 54 vs 50%), have received six or more sessions (62 vs 41%) and to have received CBT (79 vs 58%).

### Client outcomes

The mean pre- and post-treatment K-10 scores for the 1303 ATAPS clients for whom these data were available were 30.0 (SD = 7.8) and 23.0 (SD = 8.1), respectively, with a significant difference between these scores of 7.0 (p < 0.001); equivalent to a shift from ‘very high’ to ‘high’ distress. Similarly, the mean pre- and post-treatment K-10 scores for the 1326 *headspace* clients for whom these data were available were 28.7 (SD = 8.2) and 22.6 (SD = 8.9), respectively, with a significant difference between these scores of 6.0 (p < 0.001); equivalent to a shift from the upper to the lower limit of ‘high’ distress. The differences achieved by both programs are statistically significant and indicative of clinical improvement.

Table 3 displays the frequency and proportion of ATAPS and *headspace* clients across K-10 psychological distress severity categories pre- and post-treatment. The proportions of clients with low and moderate distress increased, and clients with high and very high psychological distress decreased, at post-treatment across both programs. However, a relatively greater proportion of ATAPS clients experienced ‘high’ or ‘very high’ psychological distress than *headspace* clients at both pre- and post-treatment (86 vs 79% and 52 vs 48%, respectively). A greater proportion of *headspace* clients than ATAPS clients showed a reduction in severity of symptoms across K-10 categories (68 vs 57%), with less experiencing no change (17 vs 38%) and more recording a worsening of symptoms (16 vs 6%). Findings should be interpreted in the context that the time two measurements for *headspace* clients may have represented a review rather than treatment conclusion.

**Table 3:** ATAPS and *headspace* clients’ K-10 psychological distress severity: pre- and post-treatment

K-10 category and score range	ATAPS				*headspace*			
	Pre-treatment		Post-treatment		Pre-treatment		Post-treatment	
	n	%	n	%	n	%	n	%
Low (10–15)	62	4.8	256	19.6	90	6.8	351	26.5
Moderate (16–21)	119	9.1	369	28.3	187	14.1	333	25.1
High (22–29)	411	31.5	386	29.6	411	31.0	331	25.0
Very high (30–50)	711	54.6	292	22.4	638	48.1	310	23.4

*ATAPS* Access to Allied Psychological Services, *K-10* Kessler-10

## Discussion

ATAPS and *headspace* were part of a suite of reforms (including Better Access) introduced to improve access to primary mental health care in Australia. ATAPS provided primary mental health care across the lifespan. *headspace* specifically targeted young people aged 12–25 years and additionally provided general primary care and addressed issues related to alcohol and other drugs, education and vocation.

### Access to primary mental health care

In total, 20,156 ATAPS and 17,337 *headspace* clients aged 12–25 years accessed two or more psychological services in the 3-year analysis period. The difference in the sample sizes for each program are partly due to the study’s inclusion criteria, which omitted many *headspace* clients who had access to a broader range of multi-disciplinary services, and may also be attributable to ATAPS being longer standing and having greater national coverage during the analysis period. By comparison, 194,401 people aged 15–24 years received psychological therapy and focussed psychological strategies in 2007–2009 via Better Access [28]. However, this comparison should be interpreted in the context that funding is capped for ATAPS, but not for Better Access (or *headspace*), so it is not surprising that its reach is greater than that of ATAPS and *headspace*. Furthermore, many *headspace* psychological services are funded via Better Access (57%) and few (8%) via ATAPS [21].

### Client characteristics

ATAPS and *headspace* appear to have attracted clients in the 12–25 year old age bracket who had different profiles. Proportionally, males were more likely to access *headspace* than ATAPS. This may be attributable to *headspace* providing services beyond psychological care (e.g., social, educational and vocational), which may have served to initially engage them, with psychological services subsequently provided. It is recognised that males are generally reluctant to seek mental (or any) health care [29], particularly those residing in rural and remote locations [30], which may be explained by multiple factors such as lack of services, reduced mental health literacy and attitudinal barriers (e.g., stigma, stoicism) [30–32]. However, consistent with findings from national epidemiological data indicating that of those with mental disorders, females are more likely to use mental health services than males [33], proportionally, more females than males accessed both programs.

Proportionally, more ATAPS clients were in the older age groups (18–25 years) and more *headspace* clients were in the younger age groups (12–17 years). This finding makes sense since the focus of *headspace* is early intervention. The fact that ATAPS clients (or their families) were more likely to be earning a low income may be due to the age differences observed; older clients (18–25 years) were more likely to be living independently and commencing (lower) income generating roles than their younger counterparts (12–17 years) whose recorded income was based on that of their parents/family.

ATAPS clients were more likely to reside outside of major cities and more likely to reside in outer regional/remote/very remote locations than *headspace* clients. This may be attributable to the inclusion of only the 30 fully operational *headspace* centres, which were established in the first two rounds of implementation, in the present analyses and/or ATAPS being a longer standing program with wider coverage during our analysis period. In its national upscaling, *headspace* was increasingly implemented in regional locations. Furthermore, ATAPS specifically targeted hard-to-reach or disadvantaged groups, such as people in rural locations and Indigenous people, with the specific group targeted varying from one primary health care region to another.

Consistent with their varying models of service delivery, the clinical profiles of clients of both programs varied. In accordance with the usual requirement for ATAPS clients to obtain a referral from a GP and to have a diagnosed mental disorder [34], the majority of ATAPS clients had a diagnosis of depression and/or anxiety and more severe psychological symptoms. In accordance with *headspace’s* emphasis on early intervention, its clients were less likely to have a recorded diagnosis, with a small minority considered to have a full threshold mental illness, compared to their ATAPS counterparts. Notwithstanding, a paper published using more recent *headspace* data between April 2013 and March 2014 reported that the majority of its service users presented with symptoms of anxiety and depression [19].

### Treatment characteristics

The fact that *headspace* also offered non-psychological services, which were not included in the session counts here, may explain why *headspace* clients were more likely to have received only 2–3 sessions than ATAPS clients. On the surface it appears that across both programs more clients, than not, received CBT compared with other psychological interventions (in at least one session), particularly via ATAPS. However, in reality, *headspace* delivered a range of other non-CBT psychological interventions and ATAPS allowed for limited non-CBT interventions (e.g., parent skills training, narrative therapy) in targeting hard-to-reach groups with specific needs and treatment requirements.

### Client outcomes

Based on limited available outcome data, both programs achieved statistically and clinically significant improvements in the mental health of clients. On average, the psychological distress of ATAPS clients improved from ‘very high’ to the lower end of ‘high’ and for *headspace* clients from the top of the ‘high’ range to the bottom of the ‘high’ range. Again, differences in symptom severity can be explained by the *headspace* focus on early intervention, and ATAPS clients typically requiring a diagnosis (up until July 2010 with the introduction of its Child Mental Health Service for children aged 0–11, and 12–15 years in exceptional circumstances, for whom a diagnosis was not mandatory).

## Limitations and strengths

The findings should be interpreted in the context of several limitations. Not all data across both programs could be statistically compared; however, this is at least in part explained by the difference in focus of the programs, with ATAPS typically targeting people with a mental disorder and *headspace* providing early intervention. It likely that there are a few occasions (<8%) [21] in which clients may have been represented across both programs since some ATAPS sites collaborate with *headspace* to deliver services to young people. The proportion of clients with matched pre- and post-treatment outcome data (7% for ATAPS and 8% for *headspace;* note that ATAPS has outcome data for 13% of clients when including measures other than the K-10) is not ideal, but not dissimilar to rates reported in other studies of child and adolescent mental health which report that outcome measures are rarely used more than once [35]. The low proportion of clients with matched pre- and post-treatment outcome data was a key driver for the implementation of a new data collection system across *headspace* centres in 2013. This new system and process has resulted in considerable improvements in outcome data collection [20]. Finally, because we used routinely collected data and both programs were available across the nation, it was not feasible or ethical to include a control group and therefore we cannot discount the possibility that symptom improvement might have occurred in the absence of treatment [36]. However, a recent independent evaluation of *headspace* reported that there were small improvements in the mental health of *headspace* clients relative to two matched control groups [37].

The key strength is that to our knowledge, this is the first study to directly compare these large-scale national primary mental health programs in terms of their uptake, client and treatment profiles, and client outcomes. Our findings are policy-relevant for both Australia and other developed countries considering implementing national mental (and other) health programs. The flexibilities and innovations offered via ATAPS (e.g., outreach) [11] and *headspace* (e.g., offering psychological services irrespective of diagnosis and alongside other youth health services), in conjunction with free or low-cost services, have the potential to improve access to primary mental health care by young people who are hard-to-reach (e.g., those residing in rural and remote locations or who are from low income families). Additionally, our study highlights the importance of routine data collection and monitoring of large-scale programs and the utility of minimum datasets to this end. The collection of a core set of uniform data items facilitates cross-program comparisons and at minimum should capture client characteristics, their outcomes and details of the services provided. Governments could consider the value of these issues and strategies for improving consumer access and outcomes, and consistency and compliance with data collection requirements both before and during program development and implementation.

## Conclusions

Notwithstanding the limitations, our findings demonstrated that ATAPS and *headspace* delivered free or low-cost services to a substantial number of young people who were disadvantaged and historically would not have accessed services. ATAPS reached young people who were at the older end of the 12–25 years age range (18–21 years), socioeconomically disadvantaged, and resided in rural and remote locations. Consistent with its focus on early intervention and additional provision of non-psychological services, *headspace* reached people at the younger end of the 12–25 years age range (15–17 years), providing earlier intervention, and was accessed by somewhat more males. Both programs produced overall positive clinical outcomes for their respective clients who had outcome data recorded. Our findings suggest that ATAPS and *headspace* were complementary programs, striving to improve the mental health of young Australians. Through the carefully-targeted ATAPS program, GPs can assess and direct young people to mental health care providers; through *headspace*, by raising community awareness and developing an increasingly well-recognised brand, young people and their family and friends are encouraged to self-refer early in the development of a mental health problem to access a range of mental health and general wellbeing services [19]. The recent change to implementation of both ATAPS and *headspace* through Primary Health Networks will provide a unique opportunity to ensure increasingly standardised data and improve the quantity and quality of available outcome data in order to undertake a more comprehensive comparison across programs.

## Abbreviations

ATAPS: Access to Allied Psychological Services
Better Access: Better Access to Psychiatrists, Psychologists and GP through the Medicare Benefits Schedule initiative
CBT: cognitive behavioural therapy
GP: general practitioner
*headspace*: *headspace* National Youth Mental Health Foundation Ltd
K-10: Kessler-10
NSMHWB: National Survey of Mental Health and Wellbeing
WHO: World Health Organization
WMH: World Mental Health
